# Photocatalytic Degradation of Cefixime Trihydrate by Bismuth Ferrite Nanoparticles

**DOI:** 10.3390/ma15010213

**Published:** 2021-12-28

**Authors:** Ammara Nazir, Shoomaila Latif, Syed Farooq Adil, Mufsir Kuniyil, Muhammad Imran, Mohammad Rafe Hatshan, Farah Kanwal, Baji Shaik

**Affiliations:** 1 Centre for Inorganic Chemistry, School of Chemistry, University of the Punjab, Lahore 54590, Pakistan; ammara_nazir9@yahoo.com (A.N.); imran.hons@pu.edu.pk (M.I.); 2 School of Physical Sciences, University of the Punjab, Lahore 54590, Pakistan; 3 Department of Chemistry, College of Science, King Saud University, P.O. Box 2455, Riyadh 11451, Saudi Arabia; mkuniyil@ksu.edu.sa (M.K.); mhatshan@ksu.edu.sa (M.R.H.); 4 Centre for Physical Chemistry, School of Chemistry, University of the Punjab, Lahore 54590, Pakistan; farah.chem@pu.edu.pk; 5 Department of Advanced Materials Engineering for Information and Electronics, Kyung Hee University, 1732 Deogyeong-daero, Giheung-gu, Yongin-si 446701, Gyeonggi-do, Korea; shaikbaji2@khu.ac.kr

**Keywords:** BiFeO_3_, photocatalysis, cephalosporin, sunlight

## Abstract

The present work was carried out to synthesize bismuth ferrite (BFO) nanoparticles by combustion synthesis, and to evaluate the photocatalytic activity of synthesized bismuth ferrite nanoparticles against cefixime trihydrate. BFO nanoparticles were successfully synthesized using bismuth (III) nitrate and iron (III) nitrate by a combustion synthesis method employing different types of fuels such as maltose, succinic acid, cinnamic acid, and lactose. The effects of the different types of fuels on the morphology and size of the bismuth ferrite nanoparticles were investigated. Characterization of the as-obtained bismuth ferrite nanoparticles was carried out by different techniques such as X-ray diffraction (XRD), scanning electron microscopy (SEM), Energy-Dispersive Spectroscopy (EDS), N_2_-sorption analysis, Fourier-transform infrared spectroscopy (FT-IR), and ultraviolet-visible (UV–vis) spectroscopy. Photoluminescence studies were also carried out for the various bismuth ferrite nanoparticles obtained. Degradation of cefixime trihydrate was investigated under sunlight to evaluate the photocatalytic properties of the bismuth ferrite nanoparticles, and it was found that the bismuth ferrite nanoparticles followed first-order degradation kinetics in solar irradiation in the degradation of antibiotic, cefixime trihydrate.

## 1. Introduction

Production of antibiotics takes place in large quantities due to their wide use in the treatment of humans and animals for various bacterial and fungal infections [1,2,3]. However, the excretion of unabsorbed antibiotics by humans and animals, as well as improper disposal procedures of unused antibiotics by individuals, laboratories, and factories [4,5] directly and indirectly affect the environment [6]. Furthermore, the enhanced use of antibiotics in aquaculture farming for the prevention of diseases in aquaculture products, especially fish, ends with human consumption, which leads to the unnecessary presence of antibiotics in the human body. This has several harmful impacts, especially multiple drug resistance (MDR) in a variety of microbial infections [7,8]. Hence, it is extremely important to eliminate the presence of antibiotics in water bodies.

In this regard, several nanomaterials have been reported for the photocatalytic degradation of pollutants, including antibiotics [9,10,11]. Metal oxide nanoparticles such as ZnO have been employed for the degradation of metronidazole, an antibiotic, and yielded a 98.4% removal of the antibiotic and followed pseudo-first-order degradation kinetics [12]. Song et al. further combined ZnO with Ag_2_O on Ni foam for the decomposition of antibiotics such as sulfadiazine and ciprofloxacin, which yielded decomposition of 92.56% and 99.07%, respectively [13]. Another mixed metal oxide such as a Bi_2_WO_6_-BiSI photocatalyst was employed for the degradation of tetracycline [14]. Another antibiotic, moxifloxacin, was photocatalytically degraded using CeO_2_/La_2_O_3_/TiO_2_ nanocomposites [15]. A PbMoO_4_ catalyst was employed for the degradation of ciprofloxacin, and a 100% degradation efficiency was observed [16]. Among these, one of the most commonly used antibiotics is cefixime trihydrate (CFX), which used to treat a variety of bacterial infections, including bronchitis and gonorrhea. Several photocatalysts have been reported for its degradation, such as a ZnO/GO nanocomposite, which yielded 86% CFX degradation [17]; ZnBi_2_O_4_ nanoparticles, which yielded 89% CFX degradation in sunlight within 30 min of irradiation [18]; a Fe_3_O_4_/TiO_2_ nanocomposite core-shell, which degraded CFX within 25 min [19]; and a g-C_3_N_4_ nanocomposite of Fe_3_O_4_/TiO_2_, which reportedly degraded 98 % of CFX [20]. These examples demonstrated that the degradation efficiency of antibiotics especially depended upon several surface factors of the prepared photocatalysts.

Bismuth ferrite (BFO) is a mixed metal oxide having heavier elements that are stabilized through the spin–orbit interaction [21,22,23]. BFO also possesses a small bandgap (2.5 eV), due to which it has attracted considerable attention for its photovoltaic performances [24,25,26], as well as in its applications as sensors, and in information storage and optoelectric devices [27,28,29]. Hence, the synthesis of BFO has been extensively studied using various methods such as co-precipitation [30,31], low-temperature synthesis [32,33], the sol-method [29], hydrothermal method [34], microwave hydrothermal method, solid-state reaction [35,36], rapid liquid-phase sintering technique [37], pulsed laser deposition [38], electro-spinning [39], magnetron sputtering [40], Pecchini method [41], mechanochemical synthesis [32], and combustion methods [29,42] to obtain BFO particles with destined morphologies. However, the combustion method is preferred over the other method due to various reasons such as simplicity, rapidity, and effectiveness in achieving fine and homogeneous nano-powders. Recently, various methods have been utilized to prepare BFO as a catalyst to degrade dyes, pesticides, and so on [43,44,45,46]. Different fuels have a different heat of combustion, reducing valency, and decomposition temperature, which affects the combustion reaction, morphological, and other properties of nanoparticles [42]. For instance, with higher decomposition temperatures, nucleation and growth of the particles will enhance crystallite size, and vice versa [47]. Our group has been working on the synthesis of various nanomaterials, and has been employing them in various applications, including the degradation of dyes. Hence, in continuation of the work, we proposed to prepare bismuth ferrite employing a combustion method and using several fuels such as maltose, succinic acid, cinnamic acid, and lactose; the samples obtained were labeled as BFO-M, BFO-S, BFO-C, and BFO-L, respectively. A detailed characterization of the samples obtained and photocatalytic degradation of the antibiotic cefixime trihydrate (CFX) was carried out to confirm their photocatalytic behaviors.

## 2. Experimental Methodology

### 2.1. Materials

All the chemicals used, including inorganic salts such as bismuth (III) nitrate pentahydrate (BiNO_3_.5H_2_O), iron (III) nitrate nanohydrate (FeNO_3_.9H_2_O), phosphoric acid(H_3_PO_4_), and sodium hydroxide (NaOH); and organic compounds such as methanol, succinic acid, cinnamic acid, lactose, and maltose, were of analytical grade (purity > 99.99%), and were purchased from Merck. Cefixime trihydrate was obtained from Pharmagen limited Lahore (Pakistan) and used without further purification.

### 2.2. Synthesis of Bismuth Ferrite (BFO) Nanoparticles

In a typical procedure, 0.001 M solution of bismuth nitrate and iron nitrate in a 1:1 (50 mL each) ratio were mixed, which in turn were mixed with various fuels (25 mL each) separately. The water content in the resultant mixture was evaporated on a hot plate at 80 °C until a dry slurry was obtained. This was then subjected to heating at a higher temperature (450 °C) in a furnace, until the reaction mixture boiled, foamed, caught fire, and burned with a smoldering flame, leaving a brown foam-like mass at the base of the beaker. In general, a combustion reaction ignited spontaneously, with the fuel and the nitrates from the metal nitrate precursor as the oxidizer. The resulting mass was later calcined at 600 °C for 2 h. Brown-colored powder; i.e., (BFO), was obtained and stored for photocatalytic evaluation and characterization. Bismuth ferrite (BFO) nanoparticles were prepared through combustion synthesis, using different types of organic substances such as maltose, lactose, cinnamic acid, and succinic acid as fuel, and the resulting products were labeled as BFO-M, BFO-L, BFO-C, and BFO-S, respectively.

### 2.3. Degradation Studies

Degradation of cefixime trihydrate was examined as a model reaction to study the photocatalytic activity of the synthesized BFO nanoparticles. The photocatalyst experiments were performed under sunlight at room temperature. In a typical reaction, a 50 mL solution of cefixime trihydrate (0.001 g) preloaded with 0.01 g of BFO nanoparticles was stirred for 30 min. Aliquots of the mixture were taken at a definite interval of time and analyzed using a UV–visible spectrophotometer after removing the BFO nanoparticles through centrifugation. The cefixime trihydrate degradation percentage was calculated using Equation (1) as:

(1) $$D\%=\frac{C_{o-}C_{t}}{C_{o}}\times 100$$

where *C_o_* is the initial concentration of CFX and *C_t_* is the concentration of CFX at time t.

Keeping in view of the morphological results, optimization was carried out for BFO-M. Effects of pH, catalyst dosage, cefixime dosage, and contact time were studied. Reusability and stability studies were also carried out to evaluate the degradation of cefixime under sunlight up to four cycles.

Cefixime stock solution (500 mg/L) in methanol was prepared; 0.1 M of phosphoric acid and sodium hydroxide was used to adjust the pH of the solution. At one time, the effect of one parameter was studied while keeping other parameters constant. The cefixime working solutions in the range of 1 mg/L–60 mg/L were prepared by diluting the stock solution with methanol. In a 50 mL working solution of cefixime, a certain amount (10–60 mg) of BFO-M was added. A pH in the range of (3–9) was adjusted using phosphoric acid and sodium hydroxide and measured with a pH meter. The solution was stirred on a magnetic stirrer under sunlight for 60 min. Absorbance was noted after definite intervals using a UV–visible spectrophotometer.

Degradation of cefixime trihydrate by nanoparticles was also monitored by HPLC analysis. A 10 ppm solution of cefixime trihydrate was freshly prepared in 70% methanol and used as a standard solution. A mixture of each type of BFO nanoparticle (BFO-C, BFO-S, BFO-L, and BFO-M) and cefixime trihydrate was prepared and placed under sunlight for 30 min. The sample solution was filtered and degassed by sonication before use. The mobile phase for HPLC was prepared by mixing 975 mL of water and 25 mL of tetrabutylammonium hydroxide. Phosphoric acid was used to adjust the pH to 6.5, and then 250 mL acetonitrile was added. The mobile phase was filtered and sonicated to achieve degassing. The data were analyzed by obtaining the area under the sample peaks at 254 nm.

### 2.4. Characterization Techniques

BFO nanoparticles synthesized through combustion method using different fuels were characterized by various characterization techniques. The optical absorption of BFO nanoparticles was measured at room temperature using a UV–vis spectrophotometer (Shimadzu UV–Vis 52550 spectrophotometer, Waltham, MA, USA). FTIR studies were carried out using a JASCO 4100 spectrometer to detect the possible functional groups that were useful for identifying the bonding of Bi and Fe with oxygen. Using the SEM technique, the morphologies of the BFO nanoparticles were examined. The SEM (VEGA3 TESCAN) was employed at an accelerating voltage of 12.5 kV. The energy-dispersive spectroscopy (EDS) is a very important tool for elemental analysis, and the elemental composition of the BFO was identified using this technique. The room temperature powder X-ray diffraction pattern of BFO nanoparticles calcined at 600 °C was recorded using a PANalytical X’pert (CuKα = 1.54060 A°). Details of the HPLC were: LC-20 AT, SIl,20 A HT, 100–120, 220–240 V, 50–60 Hz, 140 V/A. A pH meter (Amtast AMT12, Hong Kong, China) was used for adjusting and measuring the pH of solutions.

## 3. Results and Discussion

### 3.1. X-ray Diffraction Analysis (XRD)

**Figure 1:**
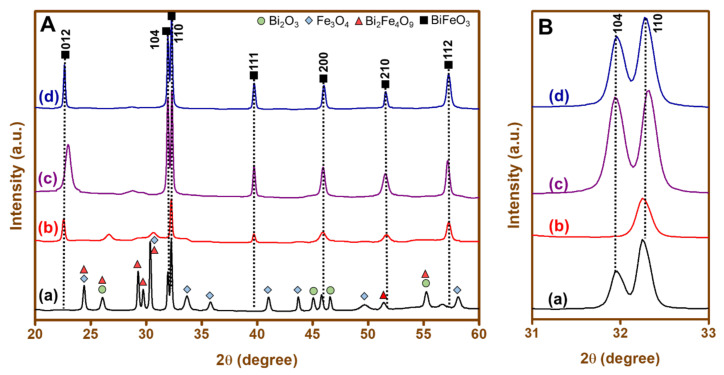
XRD pattern of (**A**) (a) BFO-C, (b) BFO-S, (c) BFO-L, and (d) BFO-M nanoparticles; (**B**) enlarged 2θ portion between 31°and 33°.

The crystalline phases of BFO nanoparticles synthesized using different fuels were identified using X-ray diffraction spectroscopy. Figure 1A displays the diffractograms obtained for BFO nanoparticles synthesized by a combustion method using different fuels such as (a) cinnamic acid, BFO-C, (b) succinic acid, BFO-S, (c) lactose, BFO-L, and (d) maltose, BFO-M, calcinated at 600 °C. The BFO-C in Figure 1A (a) exhibited mostly diffractograms corresponding to separate oxides of bismuth and iron [48]. Additionally, prominent peaks were observed for phases of other mixed metal oxides such as Bi_2_Fe_4_O_9_ [49]. However, the presence of traces of BFO nanoparticles was identified from the detected XRD pattern around 32°. The pattern exhibited in Figure 1A(b–d) were mostly in accordance with JCPDS card No. 01-072-2112, except for a slight shift in the peak at 22° for Figure 1A(c). The high intense peak at 32° indicated the orientation of BFO nanoparticles in the (110) phase direction with a rhombohedral crystal system (space group *R3c*). Additionally, the peak splitting observed at 32° confirmed the formation of the formerly mentioned crystal structure for all the BFO nanoparticles, except in the case of BFO-S (Figure 1B). For the latter nanoparticles, a phase transition could have occurred from a rhombohedral to a monoclinic or tetragonal structure, as evident from the merging of two peaks at 32° [50]. The reduced intensities of peaks corresponding to oxide impurities other than BFO nanoparticles were evidenced with the use of lactose, and completely absent with maltose fuel. Therefore, overall there was a good control on the phase purity by using different fuels, particularly when maltose was used. The obtained peaks for all four samples were found to be broad, which was due to the nanocrystallite size of the BFO. The average crystallite size, *D,* of the BFO nanoparticles was calculated by using Debye Scherer’s formula [Equation (2)]:

(2) D=kλ/βcosθ

where *k* = the constant shape factor (0.9), *λ* = the wavelength of X-rays (1.5406 A for Cu kα), *β* = the FWHM (full-width+8 at half-length maxima), and *θ* = the Bragg’s angle.

The average crystallite size of BFO nanoparticles was calculated as 48, 38, 32, and 24 nm for the samples of BFO-C, BFO-S, BFO-L, and BFO-M, respectively. These sizes were less than the period of spiral-modulated spin order, 62 nm [21]. Different crystallite sizes of BFO prepared with different fuels were in agreement with a similar trend reported for BiFeO_3_ using sucrose as fuel [42], and NiFe_2_O_4_ prepared using fuels such as polyvinyl alcohol (PVA), glycine, and urea [47]. It was established that different decomposition temperatures of fuels were responsible for different nucleation and growth of particles.

### 3.2. UV–Vis Absorbance Spectroscopy (UV-Vis)

**Figure 2:**
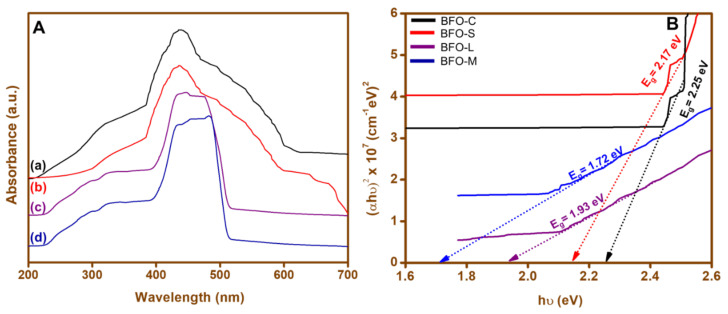
(**A**) UV–vis spectra and (**B**) band gap graph for (a) BFO-C, (b) BFO-S, (c) BFO-L, and (d) BFO-M nanoparticles.

The UV–vis absorption spectra of the BFO nanoparticles were recorded in the range of 200–700 nm at room temperature. Figure 2A shows the absorption spectra of the as-synthesized BFO nanoparticles of (a) BFO-C, (b) BFO-S, (c) BFO-L, and (d) BFO-M calcinated at 600 °C. The prepared nanoparticles showed maximum absorption in the region of 400–600 nm. The absorption maxima of BFO nanoparticles (a) BFO-C, (b) BFO-S, (c) BFO-L, and (d) BFO-M were found to be 436, 440, 482, and 504 nm, respectively, as shown in Figure 2A. The bandgap energy was determined with the Tauc model by plotting spectral dependence (αhν)^2^ over the incident photon energy hν, where α is the absorption coefficient given in Figure 2B. The figure illustrates the calculation of the bandgap energy (E_g_) using the Tauc equation, (αh ν)*^n^*= C(h ν −E_g_), where α is the absorption coefficient, h is Planck’s constant, ν is the light frequency, E_g_ is the bandgap energy, and C is a constant. The value of *n* was chosen as 2, which corresponded to a direct bandgap [51], while the value of α was calculated as follows. α = 2.303 × 10^3^ ρ*A*/*lcM*, where ρ is the theoretical density of BiFeO_3_ (8.22 g cm^−3^), *A* is the absorbance of the BiFeO_3_ nanocatalyst solution, *l* is the optical path length of the quartz cell (1 cm), *c* is the molar concentration of the suspension solution, and *M* is the molecular weight of the BiFeO_3_ nanocatalyst [52]. Different band gaps of 1.72, 1.93, 2.17, and 2.25 eV were calculated for BFO-M, BFO-L, BFO-S, and BFO-C, respectively, which might have been due to morphology and phase impurity. Moreover, these band gaps for BFO-C, BFO-S, BFO-L, and BFO-M were in the range previously reported for BiFeO_3_ (1.8–2.5 eV) [53,54].

### 3.3. Fourier-Transform Infrared Spectroscopy (FTIR)

**Figure 3:**
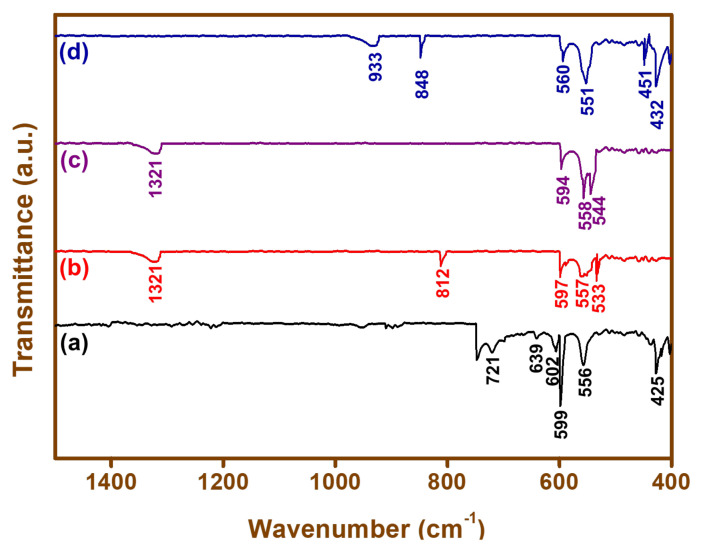
FTIR spectra of (**a**) BFO-C, (**b**) BFO-S, (**c**) BFO-L, and (**d**) BFO-M nanoparticles.

Figure 3 shows the FTIR spectra obtained for the BFO nanoparticles. Six characteristic peaks were observed in the BFO-C, five peaks were observed in the BFO-S, four peaks were observed in the BFO-L, and six peaks were observed in the BFO-M nanoparticle samples. The peaks in Figure 3a for BFO-C at the positions of 425 cm^−1^, 556 cm^−1^, 599 cm^−1^, and 602 cm^−1^ were due to stretching and bending vibrations of Fe-O bonds; and the peaks at 639 cm^−1^ and 721 cm^−1^ were due to Bi-O. In Figure 3b, the BFO-S showed peaks at 533 cm^−1^, 557 cm^−1^, and 597 cm^−1^ for (Fe-O), and at 1321 cm^−1^ for trapped nitrates. The characteristic peak at 812 cm^−1^ was due to Bi-O in BiFeO_3_ instead of Bi-O in Bi_2_O_3_, and matched with previously reported related work [55,56]. In Figure 3c, the BFO-L peaks at 544 cm^−1^, 558 cm^−1^, 594 cm^−1^, and 1321 cm^−1^ were assigned to Fe-O bending and stretching vibrations. In Figure 3d, for BFO-M only, the Fe-O peaks were found at 432 cm^−1^, 451 cm^−1^, 551 cm^−1^, and 560 cm^−1^, as well as a peak at 933 cm^−1^. The peaks at 451 cm^−1^ and 560 cm^−1^ were attributed to Fe–O stretching and O–Fe–O bending vibrations of FeO_6_ groups in perovskite BiFeO_3_, respectively, and were in agreement with the related literature [42]. We could compare our results with Ke et al., who reported that the peaks in the region of 400 to 600 cm_−1_ were the characteristics peaks for metal oxide that confirmed the synthesis of BFO [57].

### 3.4. Photoluminescence (PL)

**Figure 4:**
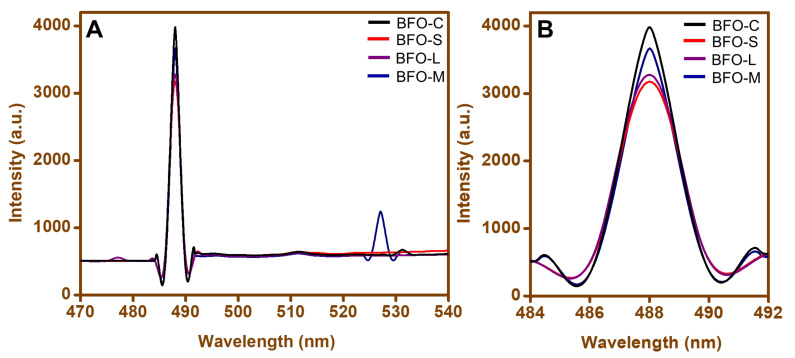
(**A**) PL spectra for (a) BFO-C, (b) BFO-S, (c) BFO-L, and (d) BFO-M nanoparticles and (**B**) an enlarged PL intensity around the 487 nm wavelength.

The PL emission spectra of the BFO nanoparticles were recorded at an excitation wavelength of 400 nm. In all the samples (BFO-C, BFO-S, BFO-L, and BFO-M), an emission peak appeared at 487 nm (Figure 4A), as reported by Gao et al. [58]. This phenomenon could be attributed to near-band-edge emission as a result of the radiative transition of photogenerated carriers (electrons–holes). An enlarged PL intensity around the 487 nm wavelength is portrayed in Figure 4B. The distinct intensities in emission peaks generated by BFO nanoparticles were attributed to the rate of recombination of photogenerated electron–hole pairs. In Figure 4A, the emission peak appeared at 527 nm for BFO-M. This smaller emission could be attributed to the presence of a shallow acceptor donor energy level below the conduction band. The shallow levels could have originated due to defects at the grain boundaries. The grain boundaries acted as a donor for electrons. Thus, charge separation enhanced the photocatalytic reaction caused by the lattice strain localized at the grain boundary.

### 3.5. Scanning Electron Microscope (SEM)

**Figure 5:**
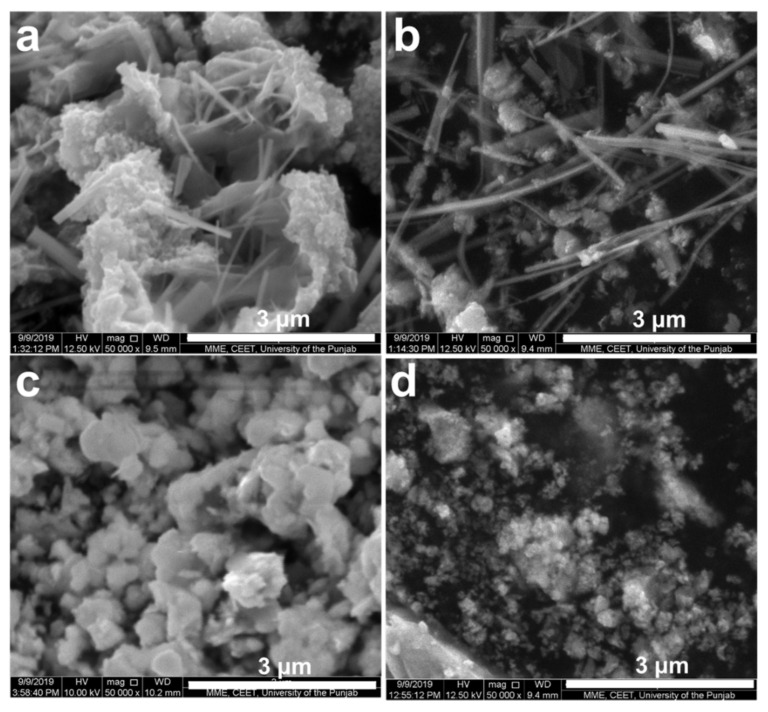
SEM images of (**a**) BFO-C, (**b**) BFO-S, (**c**) BFO-L, and (**d**) BFO-M nanoparticles.

The morphological studies of as-synthesized BFO nanoparticles were carried out with the help of scanning electron microscopy. Distinct shapes were observed with the use of different fuels for the synthesis of BFO nanoparticles. The different morphologies obtained were due to different types of fuels used. Shapes similar to rods [56] and wires [58] were observed for (a) BFO-C and (b) BFO-S nanoparticles. However, in the case of SEM images of (d) BFO-M and (c) BFO-L nanoparticles, shapes such as rods and wires were not observed, and instead only spherical particles in agglomeration were present, as seen in Figure 5c,d. The use of fuels in the process of combustion for BFO synthesis contributed a large amount of gases that were released, causing the voids and pores in the samples and ultimately affecting the morphology of the prepared BFO. The same was reported in the literature for the combustion synthesis of NiFe_2_O_4_ [47].

### 3.6. Energy-Dispersive Spectroscopy (EDS)

**Figure 6:**
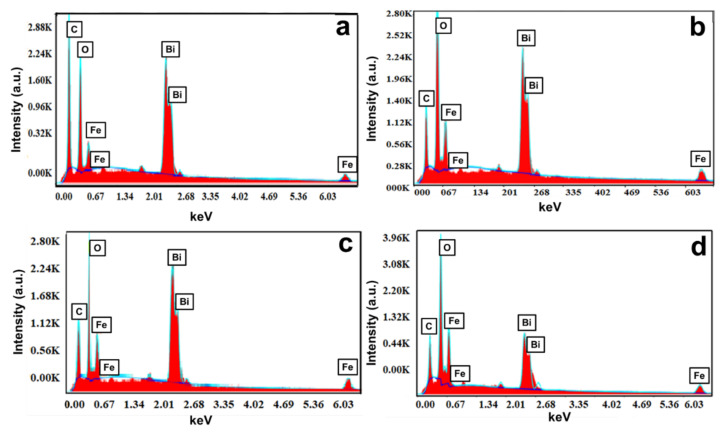
EDS micrograph of (**a**) BFO-C, (**b**) BFO-S, (**c**) BFO-L, and (**d**) BFO-M nanoparticles.

The elements present in the BFO nanomaterial samples were qualitatively and quantitatively analyzed by EDS microanalytical techniques. A graphical representation of the EDS analysis and percentage of elements by weight are plotted in Figure 6. The EDS results confirmed the presence of elements such as Bi, Fe, and O; the Fe:Bi ratio in the case of BFO-C was 1:2, in the case of BFO-S was 1.6:1, in the case of BFO-L was 1.6:1, and in the case of BFO-M was 1:0.9, in the as-synthesized BFO samples. The ratio was close to what was expected theoretically, 1:1, except for BFO-C, which involved some other secondary phase of bismuth. Overall, the elemental mapping as depicted in Figure 6 supported the successful synthesis of different fuel-based BFOs. A trace amount of carbon was also evident in the EDS images; however, the percentage was much less in the case of BFO-L and BFO-M. This carbon was either from the instrumental source or due to the release of CO_2_ from fuels during the combustion process, and was possibly trapped in voids of synthesized BFO.

### 3.7. N_2_ Sorption Studies

**Figure 7:**
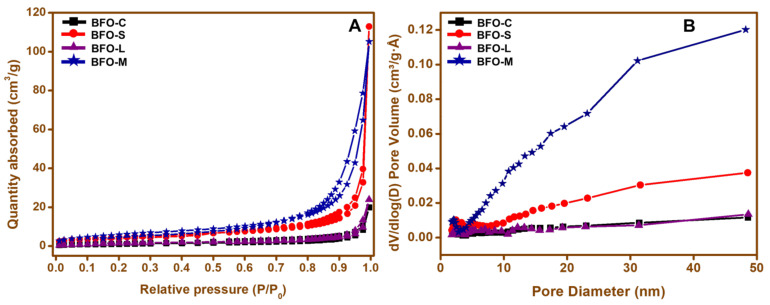
(**A**) N_2_ adsorption–desorption isotherm and (**B**) pore volume distribution graph for (a) BFO-C, (b) BFO-S, (c) BFO-L, and (d) BFO-M nanoparticles.

The N_2_ adsorption–desorption isotherm was measured for each BFO nanoparticle to investigate the effect on the Brunauer–Emmett–Teller specific surface area (BET-SSA) when using different fuels. The N_2_ adsorption–desorption isotherm and the pore size distribution for each BFO nanoparticle are represented in Figure 7. A type IV isotherm with a characteristic H3 hysteresis loop, mostly in the range of 0.8–1 (*P*/*P*_0_), was observed for all the samples (Figure 7A) [59]. The measurements of BET-SSA of 22.19, 16.21, 6.05, and 4.04 m^2^g^−1^ for BFO-M, BFO-S, BFO-L, and BFO-C nanoparticles, respectively, indicated the impact on the size of the BFO nanoparticles with the use of different fuels. It was evident from the SSA figures that the highest BET-SSA was calculated for the BFO-M nanoparticles, which were free from impure phases according to the XRD spectral data. The pore size distribution graph was estimated quantitatively with the use of the Barrett–Joyner–Halenda (BJH) method, as shown in Figure 7B [60]. As expected, the BFO-M nanoparticles exhibited a higher amount of pore distribution among all the BFO nanoparticles.

### 3.8. Degradation Studies

**Figure 8:**
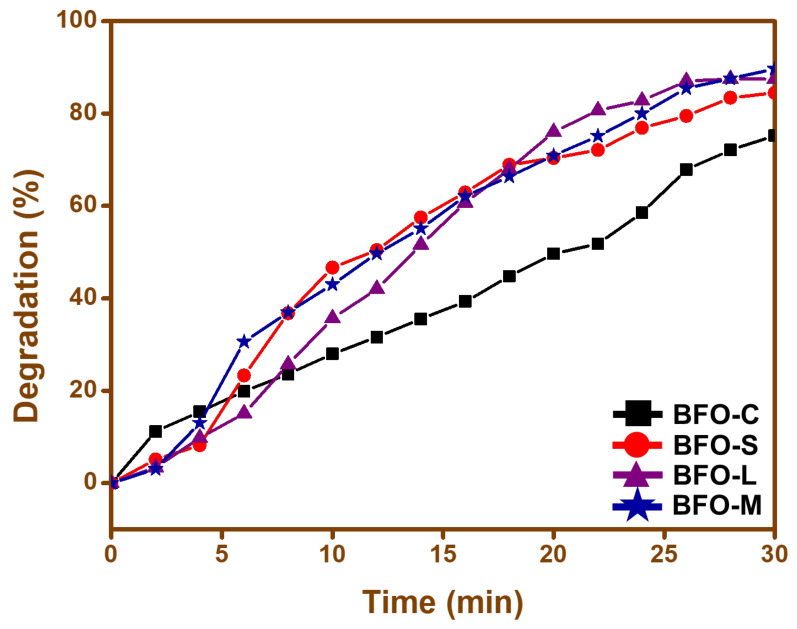
Photocatalytic activity of (**a**) BFO-C, (**b**) BFO-S, (**c**) BFO-L, and (**d**) BFO-M nanoparticles towards the degradation of the cefixime trihydrate drug.

Photodegradation of cefixime trihydrate under sunlight was used to assess the photocatalytic activity of the synthesized BFO nanoparticles. The self-degradation seemed to be insignificant, as no cefixime trihydrate was broken down when a blank experiment was conducted for 30 min without using nanoparticles in the absence of sunlight. The degradation of cefixime trihydrate using the distinct BFO nanoparticles was plotted against time in Figure 8. All the BFO nanoparticles performed well as photocatalysts. However, the degradation efficiency was dissimilar for each photocatalyst prepared. As expected, the BFO-C nanoparticles exhibited the least activity towards the degradation, with a 75% degradation rate. The rest of the nanoparticles displayed a far better activity, with more than 80% drug degradation, as shown in Figure 8. The maximum removal efficiency of cefixime trihydrate was ~90% in half an hour by the BFO-M nanoparticles, while the previously reported removal efficiency was approximately similar, about ~90%, when using TiO_2_ nanoparticles. The removal efficiency of BFO nanoparticles was better than TiO_2_ nanoparticles [61]. The degradation mechanism for the BFO nanoparticles can be given as:

(3) $$\mathrm{BFO}+h\nu \to \mathrm{BFO}\cdot +e^{-}+h^{+}$$

(4) $$h^{+}+H_{2}O\to \mathrm{OH}\cdot$$

(5) $$e^{-}+O_{2}\to O_{2}^{-}\cdot$$

(6) $${\mathrm{OH}}^{•}+O_{2}^{-•}+\mathrm{Cefixime}\;\mathrm{trihydrate}\to \mathrm{Degradation}\;\mathrm{product}$$

Nanoparticles having a larger surface area tended to show higher photo catalytic activity [62,63,64]. Spherical nanoparticles with a larger surface area showed larger photocatalytic activity as compared to rods and wires, as can be seen in Figure 8. The presence of grain boundaries, as shown in Figure 4, also supported increased photocatalytic activity in the case of maltose.

**Figure 9:**
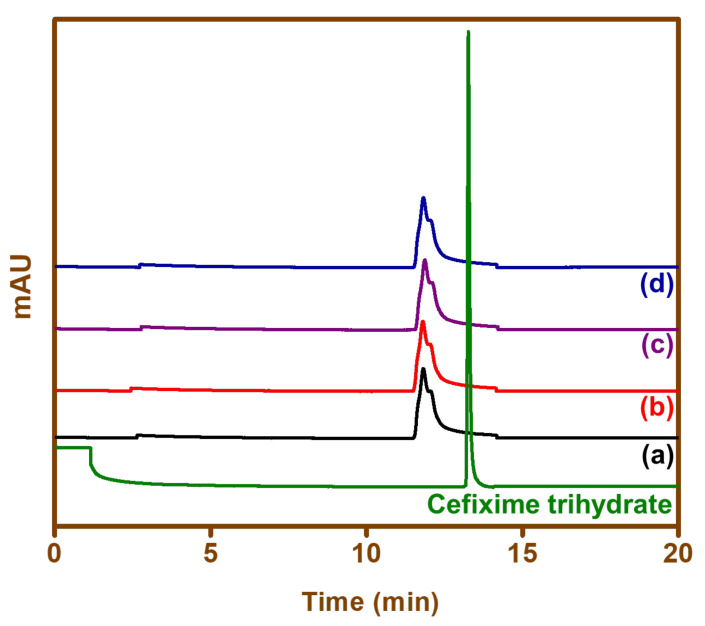
HPLC chromatograms showing cefixime trihydrate and samples of cefixime trihydrate treated with (**a**) BFO-C, (**b**) BFO-S, (**c**) BFO-L, and (**d**) BFO-M nanoparticles.

The photocatalytic heterogeneous process is normally considered to comprise different steps such as diffusion, absorption, reaction, etc. The pore distribution on the catalyst surface is effective and helpful in the diffusion of the reactant and product. In this study, the high rate of catalysis could have been due to good distribution of pores in the BFO surface, a high hydroxyl amount, and a high separation of the charge carrier. The observed retention time for cefixime trihydrate was found to be 13.12 min (Figure 9). Each sample solution was injected separately in the HPLC, and no peak appeared against the standard of cefixime trihydrate. The drug, when exposed to sunlight in the presence of different photocatalysts (BFO-C, BFO-S, BFO-L, and BFO-M), showed no corresponding peak at 13.12 min. This indicated the complete degradation of cefixime trihydrate under sunlight in the presence of the BFO nanoparticles prepared.

### 3.9. Optimization of Various Parameters

As BFO-M yielded the highest degradation percentage, it was selected as the catalyst for further optimization studies.

#### 3.9.1. Effect of the Catalyst

**Figure 10:**
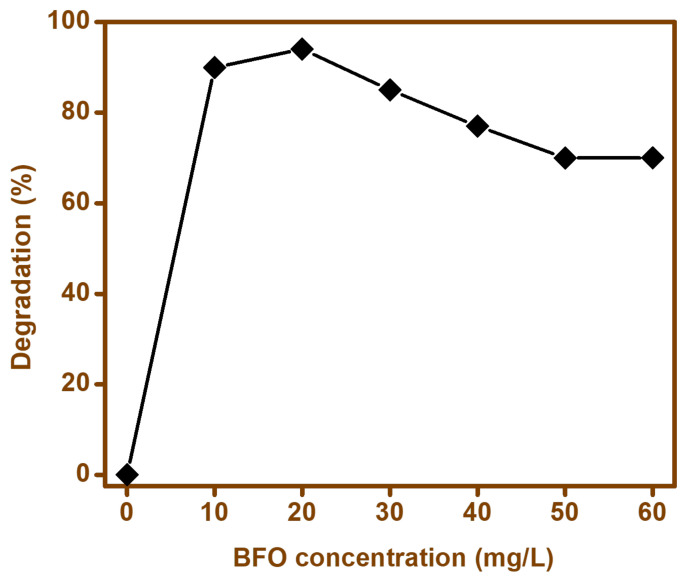
Effect of BFO concentration for the degradation of cefixime trihydrate.

The effect of catalyst loading was evaluated by varying in the rage of 1060 mg while keeping the concentration of cefixime trihydrate (1 mg/L). An increase in the degradation rate was observed when the BFO-M was increased from 10 to 20 mg; however, with a further increase in the amount of the catalyst; i.e., BFO-M, the degradation rate was observed to decrease (Figure 10). This enhancement in the degradation rate with an increase in the amount of catalyst from 10 to 20 mg could be attributed to an increase in the active sites available. However, when the amount of catalyst was further increased, a reduction in the degradation of the antibiotic was observed, which could have been due to the deactivation of the activated complex, due to the contact with the ground level molecules, hence reducing the degradation of the antibiotic in the solution. Therefore, it was concluded that when 20 mg was used, the maximum degradation was achieved [65].

#### 3.9.2. Effect of Cefixime Trihydrate

**Figure 11:**
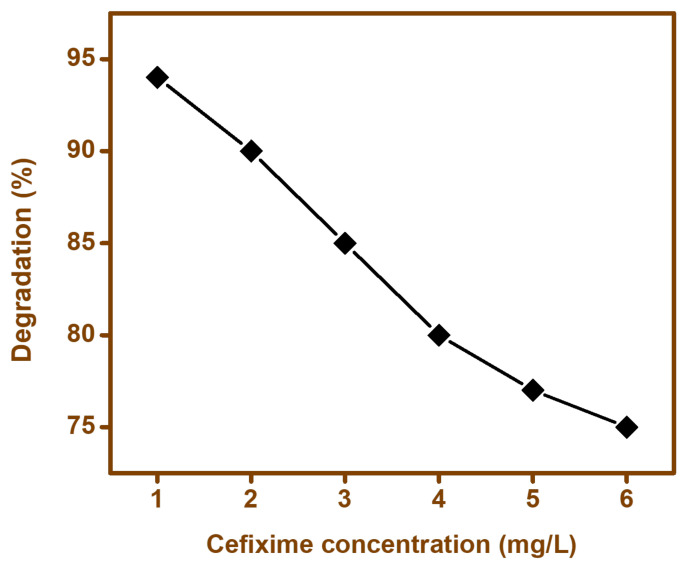
Effect of cefixime trihydrate concentration on the performance of BFO photocatalyst.

Figure 11 showed that a higher degradation rate was observed at a cefixime concentration of 1 mg/L. Cefixime was used in the range of 1 mg/L–6 mg/L. The results revealed that an increase in cefixime concentration led to a decrease in the degradation rate. It was assumed that when a solution with high concentration of contaminant was employed, a fraction of irradiated light might be absorbed by the contaminant molecules instead of the catalyst, which would result in a decrease in ^•^OH and O_2_^−^ generation, which in turn would lower the efficiency of the photocatalytic process. Additionally, the availability of the generated active species on the surface of catalysts was inadequate, as well as their probable blockage with the organic intermediates formed during the photocatalytic process, which may have resulted in a reduction in the photocatalytic degradation of the actual pollutant; i.e., cefixime trihydrate [66,67,68].

#### 3.9.3. Effect of Time

**Figure 12:**
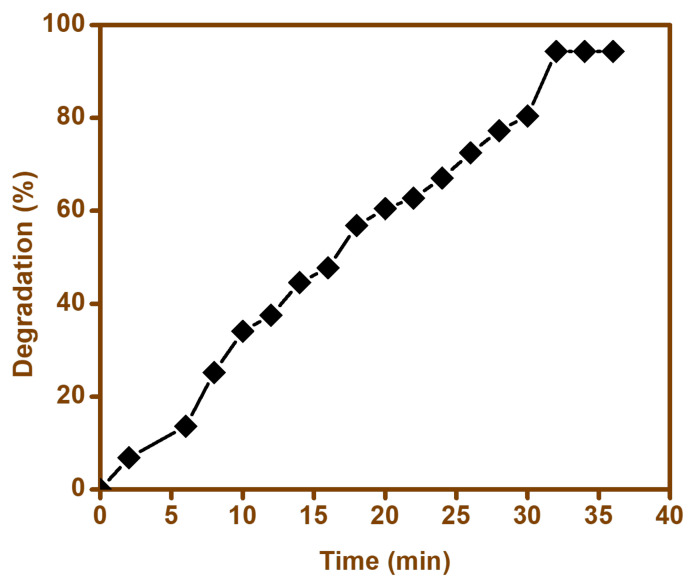
Time dependence of the degradation of cefixime trihydrate using BFO nanoparticles.

Contact time was an important factor that played a vital role in ensuring maximum efficiency of the photodegradation of the contaminant. In order to ascertain the optimum contact time for our catalyst; i.e., BFO-M, the degradation percentage for the contaminant was studied, and a sample was collected at different time intervals in order to study the degradation percentage (Figure 12). From the results obtained, we concluded that the cefixime degradation was faster during the initial 20 min of exposure time, after which the degradation efficiency reduced after 30 min, and it was then observed that no further degradation took place. Hence, it can be said that initially, cefixime was oxidized rapidly by the generated radicals; however, with passage of time, due to the formation of intermediates, the radicals generated were consumed, and the degradation efficiency was reduced [69].

#### 3.9.4. Effect of pH

**Figure 13:**
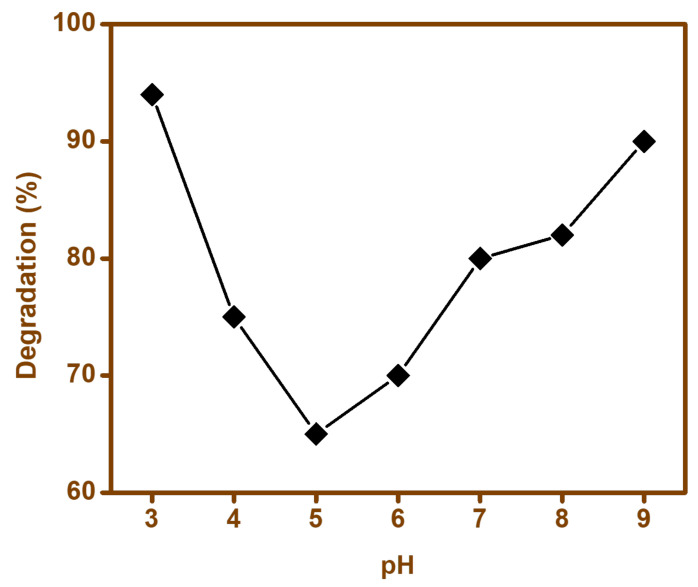
Effect of pH on the degradation of cefixime trihydrate.

The degradation of pollutants is varied by the pH of the solution, hence the degradation of cefixime was studied under various pHs (Figure 13). From the results obtained, it was evident that the degradation catalyst; i.e., BFO-M, yielded a maximum degradation efficiency at pH = 3 (94%), and a similar degradation efficiency at pH = 9 (90%). When the pH was increased from 3 to 5, the degradation efficiency was reduced; however, when it was increased up to pH = 9, the degradation efficiency improved, and was similar to the one obtained when pH = 3. The variation in the degradation efficiency with varying pH could be attributed to the electrostatic interactions between BFO charged particles and the contaminants. The pH of solution had a significant role in determining the degree of dissociation of the pollutant. This point was related to the pKa value of the pollutant, which was considered to be 2.5 for cefixime [70]. Acid–alkaline properties of the BFOs’ surface can have significant effects on their photocatalytic function [71,72,73].

**Figure 14:**
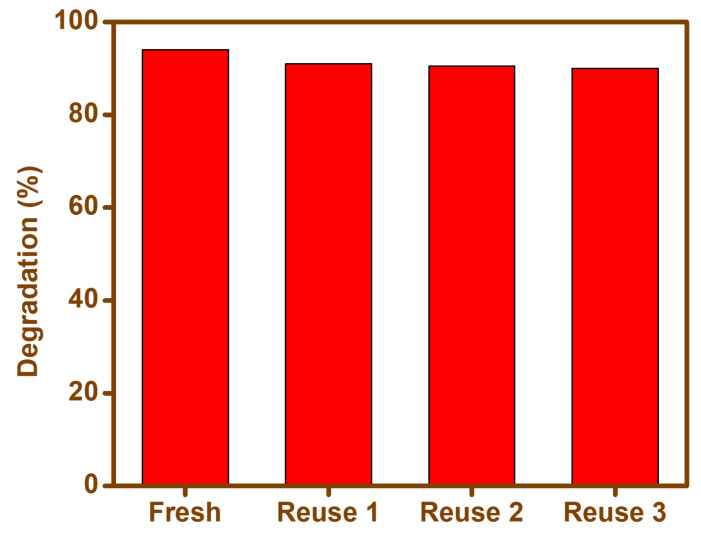
Reusability of BFO-M photocatalyst for the degradation of cefixime trihydrate.

The reusability of the BFO-M was evaluated by cefixime trihydrate degradation under visible light for up to three cycles. After each reaction, BFO-M was removed from the liquid mixture, washed twice with deionized water, and finally dried at 80 °C. The degradation rates of recovered BFO-M were slightly less (91%, 90%, and 90.5%) than first-time use under optimized conditions (94%) (Figure 14). These close values indicated a good measure of stability, and suggested the reusability of the photocatalyst without significant losses in catalytic performance.

### 3.10. Kinectics Studies

A first-order kinetics equation can usually be used for different contaminants to measure the photocatalytic reaction rate by considering the effect of intermediates and radiation [73].

**Figure 15:**
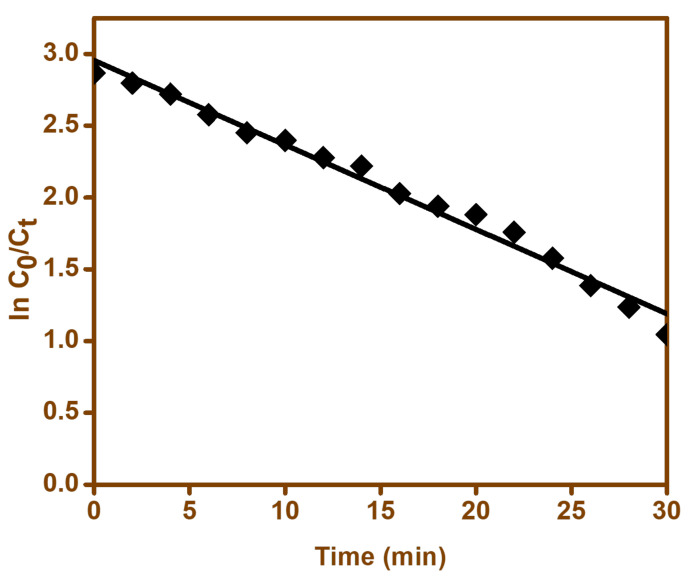
First-order kinetics model for cefixime degradation by BFO-M catalyst.

(7) lnC0Ct=kt

where *C*_0_ and *C_t_* represent the cefixime concentration at the initial time and after specific time, t, respectively. A plot of ln(*C*_0_/*C_t_*) versus irradiation time (Figure 15) gives a straight line with a slope of k. The regression analysis gave a correlation coefficient (R^2^) of 0.9838 for BFO, which displayed the good validity of the supposed first-order kinetics [74].

## 4. Conclusions

The bismuth ferrite nanoparticles with a perovskite crystal structure were successfully prepared through a combustion method using cinnamic acid, succinic acid, lactose, and maltose as different fuels. BFO nanoparticles synthesized with the use of maltose as the combustion fuel (BFO-M) were found to be the best, with a spherical shape having the lowest band gap and more photo-generated carriers at the grain boundaries. All the synthesized BFO (BFO-C, BFO-S, BFO-L, and BFO-M) nanoparticles were found to be photocatalytically active towards the degradation of cefixime trihydrate, with a highest removal efficiency of greater than 90% found when BFO-M nanoparticles under optimized conditions were employed. This process is potentially economical, as it exploits the naturally available and abundant energy source for water treatment.

## Data Availability

Data are contained within the article.
